# SNP marker discovery, linkage map construction and identification of QTLs for enhanced salinity tolerance in field pea (*Pisum sativum* L.)

**DOI:** 10.1186/1471-2229-13-161

**Published:** 2013-10-17

**Authors:** Antonio Leonforte, Shimna Sudheesh, Noel OI Cogan, Philip A Salisbury, Marc E Nicolas, Michael Materne, John W Forster, Sukhjiwan Kaur

**Affiliations:** 1 Department of Environment and Primary Industries, Biosciences Research Division, Grains Innovation Park, PMB 260, Horsham, VIC 3401, Australia; 2 Melbourne School of Land and Environment, University of Melbourne, Melbourne, VIC 3010, Australia; 3 Department of Environment and Primary Industries, Biosciences Research Division, AgriBio, Centre for AgriBioscience, 5 Ring Road, La Trobe University Research and Development Park, Bundoora, VIC 3083, Australia; 4 La Trobe University, Bundoora, VIC 3086, Australia

**Keywords:** Grain legume, Genetic marker, Trait dissection, Comparative genomics, Abiotic stress, Breeding

## Abstract

**Background:**

Field pea (*Pisum sativum* L.) is a self-pollinating, diploid, cool-season food legume. Crop production is constrained by multiple biotic and abiotic stress factors, including salinity, that cause reduced growth and yield. Recent advances in genomics have permitted the development of low-cost high-throughput genotyping systems, allowing the construction of saturated genetic linkage maps for identification of quantitative trait loci (QTLs) associated with traits of interest. Genetic markers in close linkage with the relevant genomic regions may then be implemented in varietal improvement programs.

**Results:**

In this study, single nucleotide polymorphism (SNP) markers associated with expressed sequence tags (ESTs) were developed and used to generate comprehensive linkage maps for field pea. From a set of 36,188 variant nucleotide positions detected through *in silico* analysis, 768 were selected for genotyping of a recombinant inbred line (RIL) population. A total of 705 SNPs (91.7%) successfully detected segregating polymorphisms. In addition to SNPs, genomic and EST-derived simple sequence repeats (SSRs) were assigned to the genetic map in order to obtain an evenly distributed genome-wide coverage. Sequences associated with the mapped molecular markers were used for comparative genomic analysis with other legume species. Higher levels of conserved synteny were observed with the genomes of *Medicago truncatula* Gaertn. and chickpea (*Cicer arietinum* L.) than with soybean (*Glycine max* [L.] Merr.), *Lotus japonicus* L. and pigeon pea (*Cajanus cajan* [L.] Millsp.)*.* Parents and RIL progeny were screened at the seedling growth stage for responses to salinity stress, imposed by addition of NaCl in the watering solution at a concentration of 18 dS m^-1.^ Salinity-induced symptoms showed normal distribution, and the severity of the symptoms increased over time. QTLs for salinity tolerance were identified on linkage groups Ps III and VII, with flanking SNP markers suitable for selection of resistant cultivars. Comparison of sequences underpinning these SNP markers to the *M. truncatula* genome defined genomic regions containing candidate genes associated with saline stress tolerance*.*

**Conclusion:**

The SNP assays and associated genetic linkage maps developed in this study permitted identification of salinity tolerance QTLs and candidate genes. This constitutes an important set of tools for marker-assisted selection (MAS) programs aimed at performance enhancement of field pea cultivars.

## Background

Field pea (*Pisum sativum* L.) is widely cultivated on a global basis as an important legume crop for human dietary protein intake and livestock forage nutrition [1]. Field pea is especially beneficial in crop rotations with cereals in order to provide disease breaks and for provision of soil nitrogen [2].

Development of sustainable high-yielding varieties that persist under biotic and abiotic stresses is a prerequisite for meeting the food requirements of a growing world population. Molecular breeding strategies have been adopted for crop improvement programs in several crops, including legumes such as soybean and common bean [3], and are suitable for application in field pea. Most breeding gains for grain yield in field pea have been achieved by optimisation of crop architecture (i.e. reduced internode length), harvest index and phenology traits with growing season length and rainfall [4,5]. Breeding practices have also primarily focused on pyramiding genes for resistance to important fungal diseases such as ascochyta blight, powdery and downy mildew, and viruses such as pea seed-borne mosaic virus (PSbMV) and bean leaf roll virus (BLRV). However, comparatively little effort has been directed towards improvement of physiologically complex and putatively multigenic traits such as tolerance to salinity stress [6].

Genetic improvement for complex traits will be facilitated by new genomic tools through the identification and selection of preferred genes. For legume crops, only limited genomic resources were available until recently, so MAS adoption has been slow [6]. However, advances in DNA sequencing and genotyping technologies have recently delivered large-scale transcriptome sequence data sets for field pea [7,8]. These data can be exploited for the design of DNA-based genetic markers such as SSRs and SNPs, supporting linkage mapping, analysis of genetic diversity, trait-dissection [9,10], as well as gene-tagging for MAS [11].

For pea, a large number of genetic linkage maps have been developed previously [10,12-18]. SSR markers are generally co-dominant in nature and highly polymorphic, and have been extensively used for pedigree analysis in crop breeding and genetics research [6]. SNPs are highly prevalent, usually biallelic and co-dominant in nature, sequence-tagged, and amenable to development of low-cost multiplexed marker assays that can provide sufficiently dense genome coverage for the dissection of complex traits [19,20]. A number of methods have been developed for SNP detection. Medium to high-throughput array-based SNP genotyping systems are now available, depending on the number of samples and markers to be analysed, such as GoldenGate**®** and Infinium from Illumina Inc., SNPStream from Beckman Coulter, and GeneChip from Affymetrix [18].

In order to understand complex biological processes in plants, comparative genetic analysis with model species has been used extensively. In concert with extensive genomic resources that are available for a number of species of the legume sub-family Papilionoideae (e.g. *M. truncatula* [http://www.medicago.org]*, L. japonicus*[21]*,* chickpea [22], soybean [23] and pigeon pea [24]), such analysis provides opportunities for translational genomics to assist breeding of other, less well-studied crop legumes, such as field pea.

Soil salinity is a serious global problem due to limitation of plant growth and reduced crop yield [25]. Salinity tolerance in field pea has become increasingly important in Australia due to a geographical shift of crop production towards environments characterised by shorter seasons, greater water limitation and marginal soils with higher transient soil salinity [26]. Large effects of salinity and sodicity are predominantly due to levels of the Na^+^ cation, and in Australia, are commonly associated with highly alkaline (pH > 8.5) soils [27,28]. In combination, these factors can cause nutrient (Fe, K) deficiencies and soil toxicities (such as to elevated levels of boron) that limit growth and grain yield potential. For field pea, relatively high and heritable genetic tolerances to Fe deficiency [29] and boron toxicity [30-32] have been identified. In terms of salinity tolerance, preliminary studies based on biomass reduction indicated that field pea is significantly more sensitive than other commonly cultivated Australian broad-acre crops such as barley [33,34], wheat [35] and canola [36], due to a low salinity threshold level [37] in pea. In comparison to other legumes, in contrast, pea [38-41], as well as faba bean [42], appear more tolerant than chickpea [43] and lentil [44].

Research on other major dry-land crops such as wheat [45] has demonstrated the difficulty of using yield-based response measurements from field studies as a measure of salinity tolerance, due to the complexity of interactions with other stress factors such as high pH and boron, Na^+^ variability in the soil profile, and differential responses according to both growth stage and genotype. However, low-cost and reliable pot-based glasshouse screening methodologies have been developed for a range of crops, including pea [41], which can be used to identify useful variation at the seedling stage for breeding purposes. Considerable potential for genetic improvement appears to be available, on the basis of the outcome of screening experiments [41,46]. Identification and marker-tagging of genomic regions containing QTLs for aspects of salinity stress tolerance would hence highly facilitate the targeted introgression of this trait into otherwise unadapted germplasm.

The objectives of the present study were: development and characterisation of novel SNP markers and characterisation of existing SSR markers; construction of an SSR- and SNP-based linkage map for a field pea population varying for salinity tolerance; comparative genetic analysis between field pea and other legumes of the sub-family Papilionoideae; and identification of genomic regions and molecular genetic markers associated with salinity tolerance in field pea.

## Methods

### Plant material and DNA extraction

Crosses were made between single genotypes of cultivar Kaspa (salinity sensitive), and Parafield (moderately tolerant). The crosses were performed at DEPI-Horsham in 2007 and F_2_ generation progeny were produced. Single seed descent was undertaken from F_2_ progeny-derived genotypes for 4 generations in the glasshouse from 2008 to 2011. The resulting F_6_ mapping population consisted of 134 RILs.

Frozen leaf tissue from each progeny genotype was ground using a Mixer Mill 300 (Retsch®, Haan, Germany), and genomic DNA was extracted using the DNeasy® 96 Plant Kit (QIAGEN, Hilden, Germany). DNA was resuspended in 1 x TE buffer to a concentration of 50 ng/μl and stored at -20°C.

### SNP discovery and validation

Putative SNPs were identified from transcriptome sequence data [8] using Next*GENe* software v1.96 (SoftGenetics, State College, PA, USA). Based on alignment of high-quality sequences from four genotypes (including Kaspa and Parafield [8]) with the consensus reference (obtained as a result of *de novo* assembly), all base variants were identified. All insertion and deletion (indel) variants were excluded from further analysis. Subsequently, high-confidence SNPs were filtered using the following criteria: (1) base variants in homozygous condition within each genotype; (2) read-coverage equal to or greater than 4; and (3) absence of any other base variants within 20 bp segments flanking each SNP.

A sub-set of 48 SNPs was selected for experimental validation by Sanger sequencing. Primer pairs were designed using Sequencher 4.7 (Gene Codes Corporation, USA) and OligoCalc: Oligonucleotide Properties Calculator (http://www.basic.northwestern.edu/biotools/oligocalc.html). PCR reactions contained 10 ng of genomic DNA in a 12 μl reaction with 5 μM of each primer pair. The amplification conditions were as follows: a hot start at 94°C for 15 min, followed by 35 cycles of 95°C for 30 s, 50°C for 30 s and 72°C for 1 min, and a final elongation step at 72°C for 7 min. PCR products were purified in a 15 μl reaction containing 0.5 U exonuclease I (New England Biolabs), 0.5 U shrimp alkaline phosphatase (USB-VWR International, Pennsylvania, USA) and 5 μl of PCR product. Sequencing reactions were performed in a total volume of 7.5 μl, each reaction contained 3.2 μM primer, BigDye® Terminator v3.1 (Life Technologies Australia Pty Ltd, Victoria, Australia), BigDye® sequencing buffer (Life Technologies Australia Pty Ltd, Victoria, Australia) and were subjected to cycling conditions as described in the BigDye® v.3.1 protocol. Extension products were purified by the ethanol/EDTA/sodium acetate precipitation method, resuspended in 12 μl Hi-Di™ formamide (Life Technologies Australia Pty Ltd, Victoria, Australia), and separated on the ABI3730xl automated capillary electrophoresis platform. DNA sequence analysis and alignment was performed using Sequencher 4.7, while contig assembly and the SNP validation was performed visually.

### SSR genotyping

Genomic DNA- and EST-derived SSRs [8,17] were screened on the mapping parents for polymorphism detection. Primer synthesis and PCR amplifications were performed as described previously [8,47]. PCR products were combined with the ABI GeneScan LIZ500 size standard and analysed using an ABI3730xl (Life Technologies Australia Pty Ltd, Victoria, Australia) capillary electrophoresis platform according to the manufacturer’s instructions. Allele sizes were scored using GeneMapper® 3.7 software package (Life Technologies Australia Pty Ltd).

### Framework genetic map construction and selection of maximally recombinant individuals

A framework genetic map was constructed using Joinmap® 3.0 [48] with a threshold log-of-odds (LOD) score of 3 using SSR-derived genotyping data, providing the basis for selection of maximally recombinant individuals in the mapping population using MapPop version 1.0 [49].

### SNP genotyping

A preliminary list of SNPs was selected for GoldenGate**®** primer design (Illumina Inc., San Diego, CA, USA). A designability rank score (0 to 1) was calculated for each SNP by Illumina. Finally, SNPs with designability scores between 0.7 and 1.0 were selected for development of an Illumina GoldenGate**®** oligonucleotide pool assay (OPA) for genotyping. Individuals were SNP genotyped according to the manufacturer's instructions using 250 ng of template genomic DNA. The genotyping assays were processed by the Illumina iScan reader. Automatic allele calling was achieved using the Illumina Genome Studio software v2011.1 with a GeneCall threshold of 0.20 and checking the output visually as well for the confirmation of cluster specificity.

### Genetic linkage mapping

The genetic linkage map was generated using Map Manager Software version QTXb19 [50]. Markers with a *χ*^2^ score >10 were not included in further analysis. Map distances were calculated using the Kosambi mapping function [51] at a threshold LOD score of 3. LGs were assigned on the basis of marker loci [17] in common with publicly available linkage maps of pea, and by comparison with chromosomes of *M. truncatula*[52,53]. LGs were drawn using Mapchart software v 2.2 [54].

### Comparative genome analysis

DNA sequences underlying map-assigned SSR and SNP markers were used to perform comparative analysis with genome assemblies of chickpea (NCBI, Project PRJNA175619), *M. truncatula,* v3.5 (http://www.medicago.org)*, G. max* v189 (http://www.plantgdb.org), *L. japonicus,* v2.5 (ftp://ftp.kazusa.or.jp/pub/lotus/lotus_r2.5/pseudomolecule/) and *C. cajan* v5.0 (http://www.icrisat.org/gt-bt/iipg/Genome_Manuscript.html). BLASTN was used to conduct similarity searches against each genome sequence with a threshold E-value of 10^-10^.

### Phenotypic screening

The Kaspa x Parafield RIL population was screened for response to NaCl-induced stress applied at the seedling stage. Experiments were conducted during the autumn of 2012 in a semi-controlled (polyhouse) environment at DEPI-Horsham. Screening was undertaken by sowing six plants of each RIL at equidistant spacing in 13 cm diameter pots into a sand and gravel medium (to a depth of 2 cm in two pot replications). This provided 12 plants as replicates for each RIL. The medium was composed from a 1:1 ratio of coarse river sand and 5 mm bluestone chips. Each pot was treated daily with rainwater from sowing until emergence. From 6 days post-emergence, seedlings were watered with a complete nutrient solution (i.e. nitrosol, NPK ratio 12.2: 2.9: 8.5), in addition to supplementation with a calcium source (i.e. calcium nitrate). The required NaCl concentration was tested using an electrical conductance (EC) meter and was applied at an initial rate of 3 dS m^-1^ from day 9 post-emergence. The concentration of applied NaCl was increased by 3 dS m^-1^ at each watering time to avoid abrupt osmotic shock, up to a final rate of 18 dS m^-1^, and maintained at this concentration until assessment. All watering with the nutrient and salt solution was undertaken over 3 day-intervals at a rate of 200 ml per pot applied directly to the growing medium surface. A null-salt application treatment (no added NaCl) was included for control lines (parental genotypes) and randomised in the experiment in order to eliminate effects due to other stress factors. Individual plants in each pot were assessed for symptom development (symptom score) as described previously [41] from 28 days post-emergence and thereafter on every 7^th^ day until plant death. Final plant biomass cuts were also obtained and seed set was recorded per genotype pot. Averages for plant symptom score were calculated from individual plant assessments and used to estimate genotype-specific average values for symptom score using REML spatial row-column analysis. An index was used to quantify genotypic salinity tolerance values, and to describe tolerance levels according to sensitivity based on weighted symptom scores and final biomass.

Averages for plant symptom score (calculated from individual plant assessments) and salt index were used to generate frequency distribution histograms. Narrow sense heritabilities (*h*^*2*^) were calculated for the trait by considering the spatial trends in the experiment using best linear unbiased prediction (BLUP) analysis.

### QTL analysis and candidate gene selection

QTL detection was conducted using MapManager QTX software version QTXb19. Marker regression analysis was initially performed to identify markers significantly associated with trait variation (LOD threshold = 3). Simple interval mapping (SIM) and composite interval mapping (CIM) methods were used to identify and confirm QTLs associated with salt tolerance. The sequences underpinning SNP loci flanking the QTL-containing intervals were BLAST analysed against the *M. truncatula* genome to identify genomic regions containing putative candidate genes.

## Results

### SNP discovery and validation

A total of 36,188 putative SNPs were identified from comparison of transcriptome reads obtained from the mapping parents against the EST sequence database. An average frequency of 1.85 SNPs per kb between two haplotypes was observed. A preliminary set of 21,000 SNPs were selected following elimination of indels. After further filtration based on the criteria of homozygous status and absence of other known SNPs in the vicinity, a sub-set of 956 high quality SNPs was obtained. Of these, a total of 953 satisfied the required primer design criteria and a final sub-set of 768 SNP loci with a designability rank of 1 was selected for GoldenGate**®** assay.

Analysis of nucleotide variation revealed that transition substitutions were more predominant (2:1) than transversions. The two most common SNP variants were A/G and C/T, representing 36% and 32% of all changes, respectively. The other SNP variants (T/G, C/G, A/C and A/T) accounted for less than 10% of the total (Additional file 1). A subset of 48 SNP loci was verified through Sanger sequencing prior to 768-plex SNP OPA synthesis (Additional file 2), of which 45 were concordant with prediction (Additional file 3).

### Framework genetic map construction and selection of maximally recombinant individuals

A total of 96 of 240 genomic DNA-derived SSRs and EST-SSRs (40%) revealed polymorphism between the parental genotypes, of which 78 were selected for screening on the mapping population on the basis of consistency of amplification. A sub-set of 47 SSR markers generated data of sufficient quality to generate a framework genetic map, and 40 loci (85%) were assigned to 9 LGs. These data were then used to select 101 maximally recombinant individuals for use in bin mapping.

### SNP genotyping

A total of 768 SNPs were used to genotype the 101 selected RILs. All SNPs were visually qualified, the majority producing two major clusters in Genome Studio representing the homozygous (AA and BB) genotypic classes, but occasionally a third small cluster of heterozygous (AB) genotypes was also observed (Additional file 4). The mapping population was descended to the F_6_ level, so residual heterozygosity was expected to be low (c. 5 - 10%). A total of 705 SNPs (91.7%) produced coherent data, while those generating ambiguous cluster structures were removed from further analysis. A sub-set of 462 SNPs (65%) generated polymorphic clusters within the Kaspa x Parafield mapping population and were used for genetic linkage map construction.

### Linkage mapping

**Figure 1:**
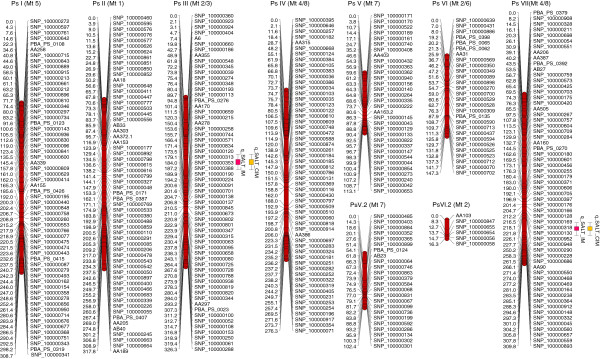
**Genetic linkage map of the Kaspa x Parafield field pea cross, showing the location of two QTLs for salinity tolerance.** The markers are shown on the right of the linkage groups and map distances between markers are indicated in cM on the left. For presentation purposes, only one of a set of co-located genetic markers are shown on the map.

A total of 73 markers (13.5%) were excluded from linkage analysis due to excessive heterozygosity, missing data, skewed segregation or ambiguity. A final set of 467 markers (53 SSRs and 414 SNPs) was used for linkage map construction. A small proportion of markers were ungrouped, such that 458 (98%), comprising 48 SSRs and 410 SNPs (Table 1) were assigned to 9 LGs (Additional file 5). The estimated cumulative total map length was 1916 cM with an average inter-locus interval of 4.2 cM (Figure 1; Table 2). LG identity and orientation were determined by comparison with the *M. truncatula* genome, as well as from the use of previously map-assigned SSRs as anchoring markers.

**Table 1:** Total number of markers analysed, tested for polymorphism and assigned to genetic linkage map locations

**Marker type**	**Total number of markers**	**Polymorphic markers**	**Mapped markers**
Genomic DNA-derived SSR	144	54	30
EST-SSR	96	24	18
SNP	768	462	410
**Total markers**	1008	540	458

**Table 2:** Marker distribution over the LGs of the Kaspa x Parafield map

**LGs**	**Predicted pea chromosome**	**Length (cM)**	**Number of mapped markers**	**Average marker density (cM)**
LG 1	Ps VII	309	87	3.6
LG 2	Ps III	326	78	4.2
LG 3	Ps I	309	69	4.5
LG 4.1	Ps V	113	35	3.2
LG 4.2	Ps V	102	25	4.1
LG 5.1	Ps VI	147	29	5.1
LG 5.2	Ps VI	16	6	2.7
LG 6	Ps IV	276	63	4.4
LG 7	Ps II	318	66	4.8
**Total**		1916	458	4.2

### Comparative genome analysis

Corresponding DNA sequences were available for 310 of 458 of the mapped loci (15 EST-SSRs and 295 SNPs), of which 307 detected significant sequence similarity matches to at least one of the reference genome sequences, and 130 sequences displayed similarity to sequences in all five genomes.

**Figure 2:**
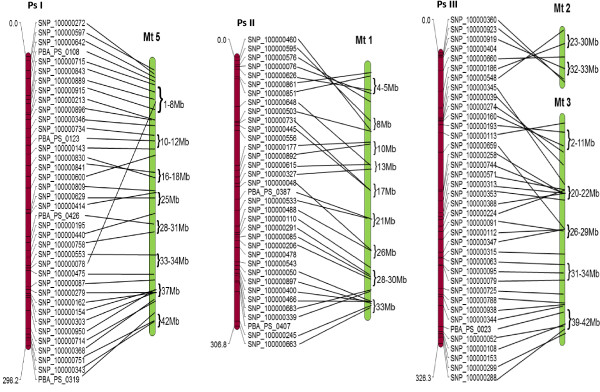
**Schematic representation of syntenic relationships between field pea (LGs PsI - III) and the *M. truncatula *genome*.*** LGs or chromosomes are shaded in different colours for presentation purposes. The red-shaded LGs are from field pea, and the green chromosomes are from *M. truncatula.* The lines represent the corresponding positions of orthologous sequences.

**Figure 3:**
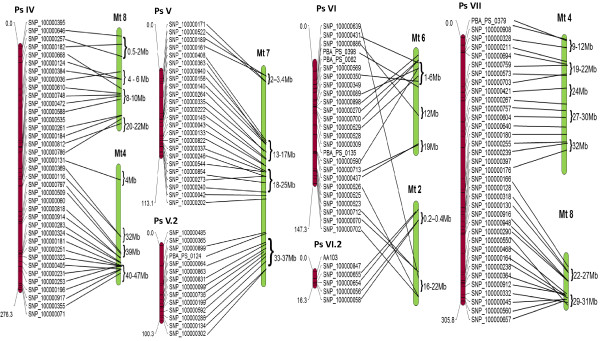
**Schematic representation of syntenic relationships between field pea (LGs PsIV - VII) and the *M. truncatula *genome.** Details are as for Figure 2.

Comparison of the field pea map with the chickpea genome revealed the highest number of matches (301: 97%) (Additional file 6). The syntenic relationships related each of field pea chromosomes Ps II, Ps IV, Ps V , V.2, and Ps VII to chickpea pseudomolecules Ca4, Ca7, Ca3 and Ca6, respectively. Some LGs containing blocks syntenic to more than one Ca group were also observed. Field pea - *M. truncatula* macrosynteny was observed for 292 (94%) sequences. Among *M. truncatula* chromosomes, Mt5, 1, 3, and 7 exhibited synteny and colinearity with pea linkage groups Ps I, Ps II, Ps III and Ps V respectively (Figures 2 and 3). Conversely, Mt2 and 6 contained the lowest number of field pea orthologues, revealing more complex relationships with PsLGs.

Despite a large number of matches (294) between field pea and soybean sequences*,* significant chromosomal rearrangements were observed between the two genomes, such that each PsLG exhibited substantial synteny with more than one soybean chromosome. Comparison with *L. japonicus* identified 226 (73%) matches with segmental syntenic blocks rather than whole chromosomal relationships. Field pea – pigeon pea synteny analysis revealed the lowest number of matches (183), short conserved regions being distributed across different chromosomes. In most instances, CcLGs were inverted in order in comparison to PsLGs, apart from CcLG 2 and 11.

**Figure 4:**
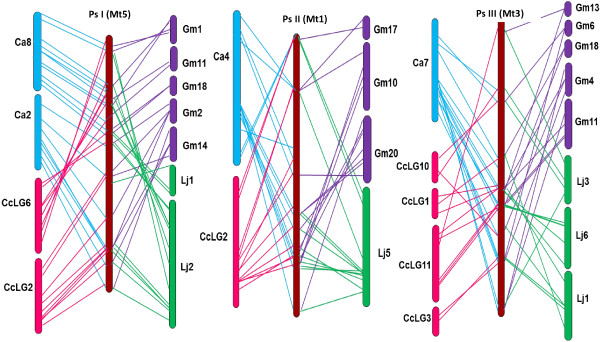
**Syntenic relationships of field pea (LGs PsI -III) with other legume genomes.** LGs or chromosomes are shaded in different colours for visualisation purposes. The details of colour codes are as follows, blue -chickpea, pink – pigeon pea, violet - soybean, green – *L. japonicus* and brown – *M. truncatula*. Coloured lines represent the corresponding positions of the orthologous sequences in field pea.

**Figure 5:**
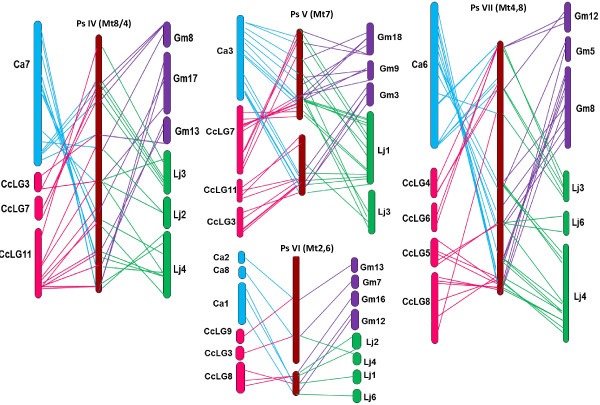
**Syntenic relationship of field pea (LGs PsIV - VII) with other legume genomes.** Details are as for Figure 4.

The 130 common orthologous sequences were used to further analyse and confirm the degree of genome conservation (Figures 4 and 5). For most PsLGs, only one or two corresponding chromosomes were identified for chickpea and *M. truncatula*, but complex relationships were observed with *L. japonicus,* pigeon pea and soybean, consistent with the pair-wise comparisons. The exception to these general patterns was Ps VI, which displayed complex relationships in all instances.

### Phenotypic analysis, QTL detection and candidate gene selection

Plant symptom response data from salinity screening of the RIL population at the seedling stage indicated that variation for tolerance was normally distributed (Additional files 7 and 8), and therefore likely to be controlled by multiple genes. The estimated narrow sense heritability (*h*^*2*^) for salt index was 0.55. Two different phenotypic measurements, including salt index and mean symptom score (average of symptom scores obtained at up to 35 days) were used to detect salt tolerance QTLs (Figure 1), with LOD scores of 3.2 (salt index) and 2.5 (symptom score) as minimum significance levels. Two QTLs were identified on Ps III and Ps VII, explaining 12% and 19% of phenotypic variance (V_p_) for salt index score, and 12% and 17% for the symptom score, respectively (Table 3). QTL analysis was also performed using symptom scores obtained at different time points (day 7, 14, 21, 35), which identified the same QTL locations and accounted for similar proportions of V_p_ (data not shown). The phenotypic data for symptom scores obtained at day 42, 49, 56 deviated from normality, and was consequently not used for QTL analysis based on mean symptom score.

**Table 3:** Identification of QTLs for salt tolerance on the Kaspa x Parafield genetic map based on CIM

**Trait**	**Flanking markers**	**Linkage group**	**Position (cM)**	**LOD threshold**	**Max LOD score**	**Phenotypic variance (%)**
Salt index_QTL 1	SNP_100000313	Ps III	179 - 184	3.2	3.9	12
	SNP_100000353					
Salt index_QTL 2	SNP_100000318	Ps VII	218 - 222	3.2	4.7	19
	SNP_100000130					
Symptom score_QTL 1	SNP_100000313	Ps III	179 - 184	2.5	3.9	12
	SNP_100000353					
Symptom score_QTL 2	SNP_100000318	Ps VII	218 - 222	2.5	5.9	17
	SNP_100000130					

**Figure 6:**
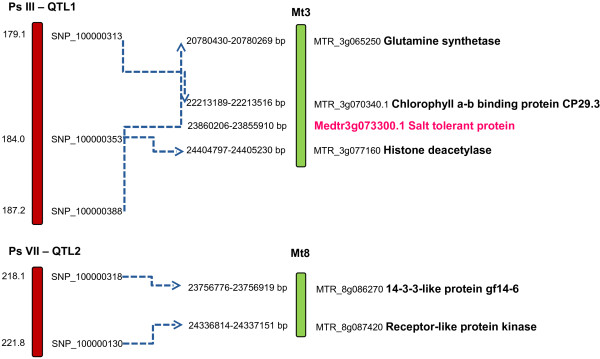
**Syntenic relationships between salt tolerance QTL-containing regions of the field pea genetic map and the *M. truncatula *genome, indicating candidate gene locations.** LGs or chromosomes are shaded in different colours for presentation purposes. The red-shaded LGs are from field pea, and the green chromosomes are from *M. truncatula*.

Comparison of linked marker-associated sequences to the *M. truncatula* genome directly identified candidate genes with functional annotations as receptor-like protein kinase, 14-3-3-like protein, histone deacetylase and glutamine synthetase, which have been reported as being involved in the complex salt tolerance mechanisms of plants (Figure 6). In addition, regions of the *M. truncatula* genome immediately adjacent to and within the intervals between orthologues of the linked SNP-associated sequences were examined for candidate gene presence. The Medtr3g073300.1 gene was located in the interval between field pea SNP markers SNP_100000313 and SNP_100000353, in the vicinity of Ps III-QTL1, and was annotated as a salt tolerance protein.

## Discussion

### SNP variation in field pea

SNP frequencies in plant genomes vary significantly, depending on reproductive habit (autogamous or allogamous), diversity of populations under assessment and status (coding or non-coding) of analysed regions. The SNP frequency detected in field pea in the present study is much lower than values reported for cereal crops (16.5 SNPs per kb in wheat, 4.2 SNPs per kb in rice [55]), but similar to those for other legumes (0.9 SNPs per kb in cowpea [56], 1.96 SNPs per kb in *M. truncatula*[57] and 2.06 SNPs per kb in soybean [58]).

The patterns of nucleotide substitution showed A/G and C/T to be the most common base changes, in agreement with previous studies of legume species such as white clover [59] and chickpea [60]. The high proportion of C/T transitions are likely to be partially due to deamination of 5-methylcytosine reactions, which occurs frequently over evolutionary time, particularly at CpG dinucleotides [61].

The effectiveness and suitability of GoldenGate**®** SNP assays for genotyping mapping populations and genetic resource collections of pea has been previously demonstrated [18]. The present study provides additional SNP markers that can be utilised for molecular breeding programs. The success rate for SNP genotyping (c. 91%) was comparable to previous observations made in pea (92.7%) [18] and chickpea (90.75%) [60]. Success of SNP genotyping depends on many factors including base variant selection, adjacent SNP frequency, presence of repetitive sequences, and finally, designability score. As field pea SNP discovery was based on transcriptome sequencing from multiple genotypes [8], it is not surprising that a substantial minority of markers (c. 35%) failed to detect polymorphism in the mapping family. However, inclusion of Kaspa and Parafield among the selected genotypes ensured a high frequency of success.

### Genetic linkage mapping

Several field pea linkage maps have been previously developed with successive adoption of new molecular marker technologies [10,12-17]. The linkage map constructed in the present study exhibits a regular marker distribution, but a significantly longer cumulative genetic map (1916 cM) than would be expected on the basis of typical chiasma frequency (1–2 per bivalent) at meiotic prophase. Such expansions of the pea genetic linkage map were also previously reported (1700 cM [62]; 2202.7 cM [63]). Several factors may be responsible, including the genetic constitution of different mapping populations, mapping strategies, number and type of mapped loci, the choice of mapping software and ratio between number of markers and population size [64-67].

### Comparative genome analysis

Extensive conservation of genome structure between field pea and both chickpea and *M. truncatula* was consistent with the closer phylogenetic relationship between these species than for the other legumes used in this study. In contrast to results of previous comparative genetic studies between chickpea and other legumes [68,69], substantial macrosynteny was observed in the present study.

Broad conservation of chromosome structure was observed between the 8 chromosomes of *M. truncatula* and 7 LGs of field pea, as well as evidence for evolutionary translocations [52,70]. A number of previous studies [52,53] have described high levels of conservation associated with comparisons to Mt1 and 5, moderate conservation of Mt3, 4, 7 and 8, and low levels of conservation for Mt2 and 6. Unlike other Mt chromosomes, Mt6 is short in length with a large number of repeats, low gene content (but a significant number of NBS-LRR disease resistance genes) and high heterochromatin content [71]. Ps VI, which matches Mt2 and 6, contained the least number of orthologous sequence queries, consistent with these prior studies. The situation may potentially be remedied by development of a larger cohort of markers from Ps VI. Despite a c. 10-fold difference in the genome size between *M. truncatula* and field pea [72], the extensive synteny between the two genomes suggests that whole genome duplication has not occurred in the pea lineage subsequent to evolutionary divergence from c. 40 MYA [53,73]. The larger genome size of pea could be the consequence of multiple transposition events [74]. The results of the present study have substantially extended comparative knowledge of the field pea and *M. truncatula* genomes, and such information may be used for candidate gene selection for further application to breeding programs.

In contrast, large syntenic blocks spanning entire PsLGs were absent from the comparisons with the *L. japonicus,* soybean and pigeon pea genomes*.* The former is a member of the Galegoid clade of the Papilionoideae sub-family, but more distantly related to pea than *M. truncatula* and chickpea, while the latter two are members of the Phaseoloid clade, so the observed relationships are in accord with broad phylogenetic affinities [75]. For soybean, the more limited relationships arose despite a large number of orthologous sequences, potentially also reflecting the complex paleopolyploid genome architecture of this species [76]. The field pea – *L. japonicus* comparison revealed similarities, but was frequently interrupted by chromosomal rearrangements. Similar segmental syntenic relationships were observed between *L. japonicus* and the Galegoid forage legume white clover [77], as also inferred from comparison to *M. truncatula*[78]. Extensive chromosomal rearrangements were evident between field pea and pigeon pea*,* again indicating the effects of taxonomic divergence.

### Phenotypic analysis, QTL detection and candidate gene selection

Plant response to salt tolerance is influenced by various physiological mechanisms, which are likely to be controlled by multiple genes across different environments [79]. The present study suggests a quantitative basis for seedling-induced salinity tolerance derived from adapted and high-yielding parental field pea genotypes, and a medium level of heritability, c. 45% of the variation being due to non-genetic factors. Two QTL loci were identified on Ps III and Ps VII, each accounting for moderate proportions of V_p_. Studies of different physiological traits associated with salt tolerance in *M. truncatula* identified a total of 19 putative genomic regions, the largest number of QTLs being located on Mt8 followed by Mt5, 1, 3, 4, 7, 6, and 2 [80]. A direct comparative QTL analysis could not, however, be performed due to inaccessibility of *M. truncatula* sequences associated with markers flanking the QTL intervals. However, the comparative genome analysis revealed macrosyntenic relationships between Ps III and Mt2/3, and Ps VII and Mt4/8. It is hence possible that the QTLs identified in the present study may be conserved between the Galegoid legumes.

The present study identified candidate genes associated with salt tolerance mechanisms in field pea. Histone deacetylase and glutamine synthetase have a key role in salt stress resistance in plants [81,82], while 14-3-3 proteins regulate the activities of a wide array of targets and play an important role in responses to saline stress [83]. Receptor-like protein kinases are involved in a diverse range of processes including biotic/abiotic stress response [84]. Furthermore, the salt tolerance protein (STO) was identified as one of the gene products involved in the regulation of the internal Na^+^/K^+^ ratio, an essential process for salinity tolerance [85]. The genes identified within the QTL-containing regions are therefore plausible candidates, although additional studies will be required for validation.

The QTLs identified in the present study are associated with seedling growth-stage salinity tolerance. Similarly, QTLs for seedling growth tolerance have been identified in numerous grain crops, including rice [86], barley [87], soybean [88] and wheat [89]. Mechanisms related to other QTLs for growth-response occurring at germination (in tomato [90,91], rice [92], barley [93] and wheat [89]) or during reproductive development (rice [94], barley [95] and tomato [96]) are likely to be significant for field pea and warrant further investigation. The substantial variation in degree and timing of salinity-induced growth responses within and between crop species highlights complexity of the trait.

Implementation of molecular markers in MAS has rarely been achieved for physiologically complex traits such as salinity tolerance [97]. In such circumstances, breeders will need to select for varying and multiple genomic regions or response mechanisms found in different germplasm, different screening environments and within different ontogenic stages. It may therefore be necessary to quantify the adaptive nature [98] of different QTLs according to varying salinity stress, and to allocate genomic values akin to index-trait based selection. Advances in genome sequencing and genotyping capacity, especially genotyping-by-sequencing (GBS), offer the potential for genome-wide marker analysis [99] and the capacity to identify all loci contributing to a trait such as saline stress tolerance, irrespective of effect magnitude. Such data may be used to develop breeding value estimates based on all trait-linked markers, in order to identify key parental lines for targeted introgression programs.

## Conclusion

The present study describes the development of a multiplexed set of EST-derived SNPs for genetic linkage map construction in field pea. Evaluation of salt tolerance under glasshouse conditions permitted identification of two significant genomic regions. Through use of sequence-associated markers, macrosyntenic relationships were determined between field pea and five other legumes and used to predict candidate genes for salt tolerance. This information may be used for the development of linked and diagnostic polymorphisms for marker-assisted selection (MAS) of salt tolerant cultivars, based on introgression of QTL-containing genomic regions from donor to recipient germplasm. As salinity tolerance is a physiologically complex trait, future research will require evaluation in different screening environments and across varying ontogenic stages to identify additional associated genomic regions. Finally, the genetic resources generated in this study will assist other trait-dissection studies and facilitate transfer of information from related legume crops for future enhanced breeding of field pea.

## Competing interests

The authors declare that they have no competing interests.

## Authors’ contributions

AL performed population development, phenotypic assessment, data interpretation and contributed to drafting the manuscript. SS performed marker discovery, map construction, QTL analysis, comparative genomics and contributed to drafting the manuscript. NC co-conceptualised the project, contributed to data interpretation and assisted in drafting the manuscript. PS and MN co-conceptualised the project and assisted in drafting the manuscript. JF, MM and SK co-conceptualised and coordinated the project and assisted in drafting the manuscript. All authors read and approved the final manuscript.

## Supplementary Material

Additional file 1 **Percentage of SNP base variants.** This file contains a pie-chart depicting the percentages of each SNP base variant class. Click here for file

Additional file 2 **Details of the 768plex SNP-OPA design.** This file contains names and sequence information for all SNP markers used for linkage mapping. Click here for file

Additional file 3 **SNP validation using Sanger sequencing.** This file contains an example of an electropherogram generated by Sanger sequencing to demonstrate SNP validation, and showing the occurrence of two arising SNPs between different mapping family parents. Click here for file

Additional file 4 **Representative clustering patterns generated by the Illumina GoldenGate® SNP Genotyping assay.** The file contains an example of clustering patterns obtained from SNP genotyping assays on two mapping populations. The data point colour codes represent: red, AA (homozygous); blue, BB (homozygous); purple, AB (heterozygous); black, no call (missing data). A) High-quality polymorphic SNP; B) Monomorphic SNP; C) SNP with a large number of heterozygous individuals; D) Failed SNP. Click here for file

Additional file 5 **Linkage map statistics.** This file contains details of different markers (SSRs and SNPs) and their corresponding positions on different LGs. Click here for file

Additional file 6 **Synteny analysis statistics.** This file details of field pea LGs, number of marker sequences and synteny with chickpea, *M. truncatula,* soybean, *L. japonicus* and pigeon pea chromosomes. Click here for file

Additional file 7 **Frequency distribution histogram.** Frequency distribution for salinity index value and qualitative rating (T (tolerant), MT-T (moderately tolerant to tolerant), MS-S (Moderately sensitive to sensitive), S (sensitive), HS (high sensitivity) for Kaspa x Parafield RIL progeny following salinity treatment of 18 dS m^-1^. Click here for file

Additional file 8 **Frequency distribution histogram.** Frequency distribution for symptom score of Kaspa x Parafield RIL progeny at 7, 14, 21, 35, 42, 49 and 56 days post application of NaCl in watering solution at 18 dS m^-1^. Click here for file
