# Supplementary material for: SNP marker discovery, linkage map construction and identification of QTLs for enhanced salinity tolerance in field pea (Pisum sativum L.)
Source: BMC Plant Biol. 2013 Oct 17;13:161. doi: 10.1186/1471-2229-13-161 (PMC4015884; doi:10.1186/1471-2229-13-161)
Supplement: Additional file 1 — Percentage of SNP base variants. This file contains a pie-chart depicting the percentages of each SNP base variant class. [file 1471-2229-13-161-S1.pptx]

## Slide 1
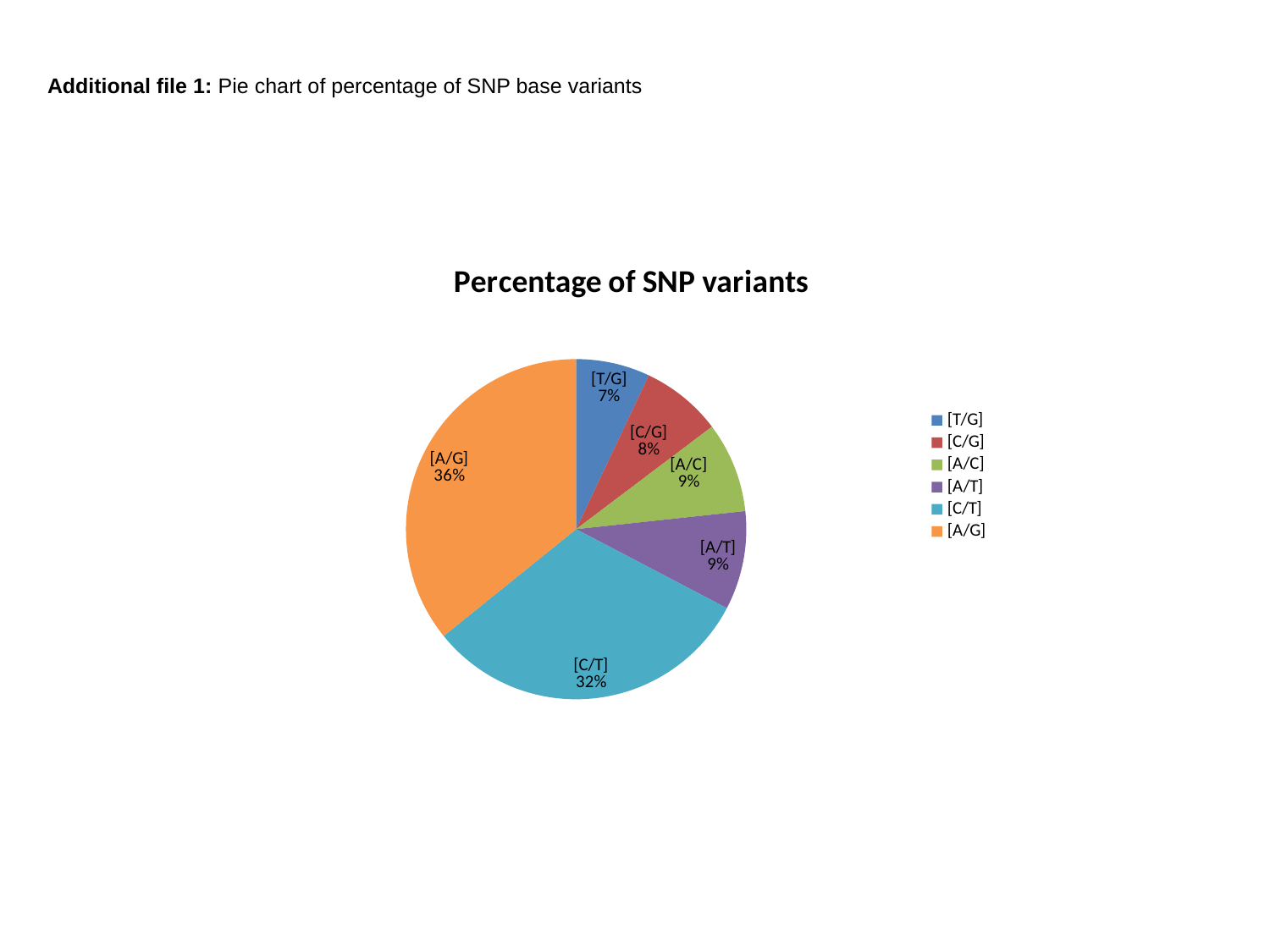

Additional file 1: Pie chart of percentage of SNP base variants
### Chart:
| Category | Percentage of SNP variants |
|---|---|
| [T/G] | 7.03125 |
| [C/G] | 7.682291666666667 |
| [A/C] | 8.59375 |
| [A/T] | 9.375 |
| [C/T] | 31.510416666666668 |
| [A/G] | 35.80729166666667 |
