# Supplementary figures and images for: SNP marker discovery, linkage map construction and identification of QTLs for enhanced salinity tolerance in field pea (Pisum sativum L.)
Source: BMC Plant Biol. 2013 Oct 17;13:161. doi: 10.1186/1471-2229-13-161 (PMC4015884; doi:10.1186/1471-2229-13-161)

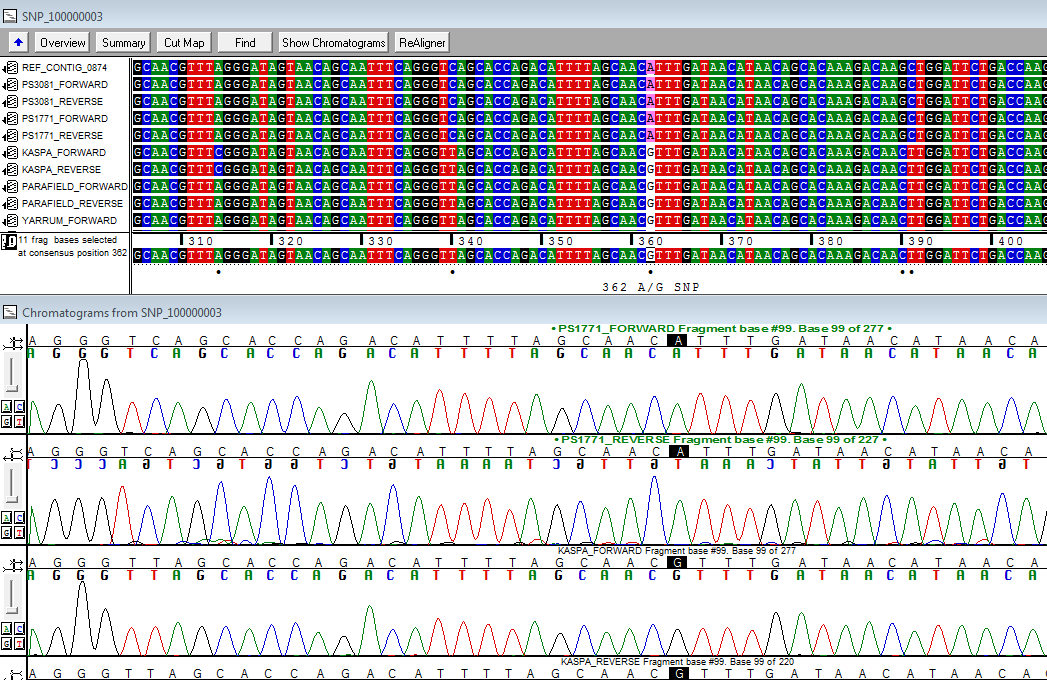

Supplement: Additional file 3 — SNP validation using Sanger sequencing. This file contains an example of an electropherogram generated by Sanger sequencing to demonstrate SNP validation, and showing the occurrence of two arising SNPs between different mapping family parents. [file 1471-2229-13-161-S3.png]
