# Supplementary material for: SNP marker discovery, linkage map construction and identification of QTLs for enhanced salinity tolerance in field pea (Pisum sativum L.)
Source: BMC Plant Biol. 2013 Oct 17;13:161. doi: 10.1186/1471-2229-13-161 (PMC4015884; doi:10.1186/1471-2229-13-161)
Supplement: Additional file 4 — Representative clustering patterns generated by the Illumina GoldenGate® SNP Genotyping assay. The file contains an example of clustering patterns obtained from SNP genotyping assays on two mapping populations. The data point colour codes represent: red, AA (homozygous); blue, BB (homozygous); purple, AB (heterozygous); black, no call (missing data). A) High-quality polymorphic SNP; B) Monomorphic SNP; C) SNP with a large number of heterozygous individuals; D) Failed SNP. [file 1471-2229-13-161-S4.pptx]

## Slide 1
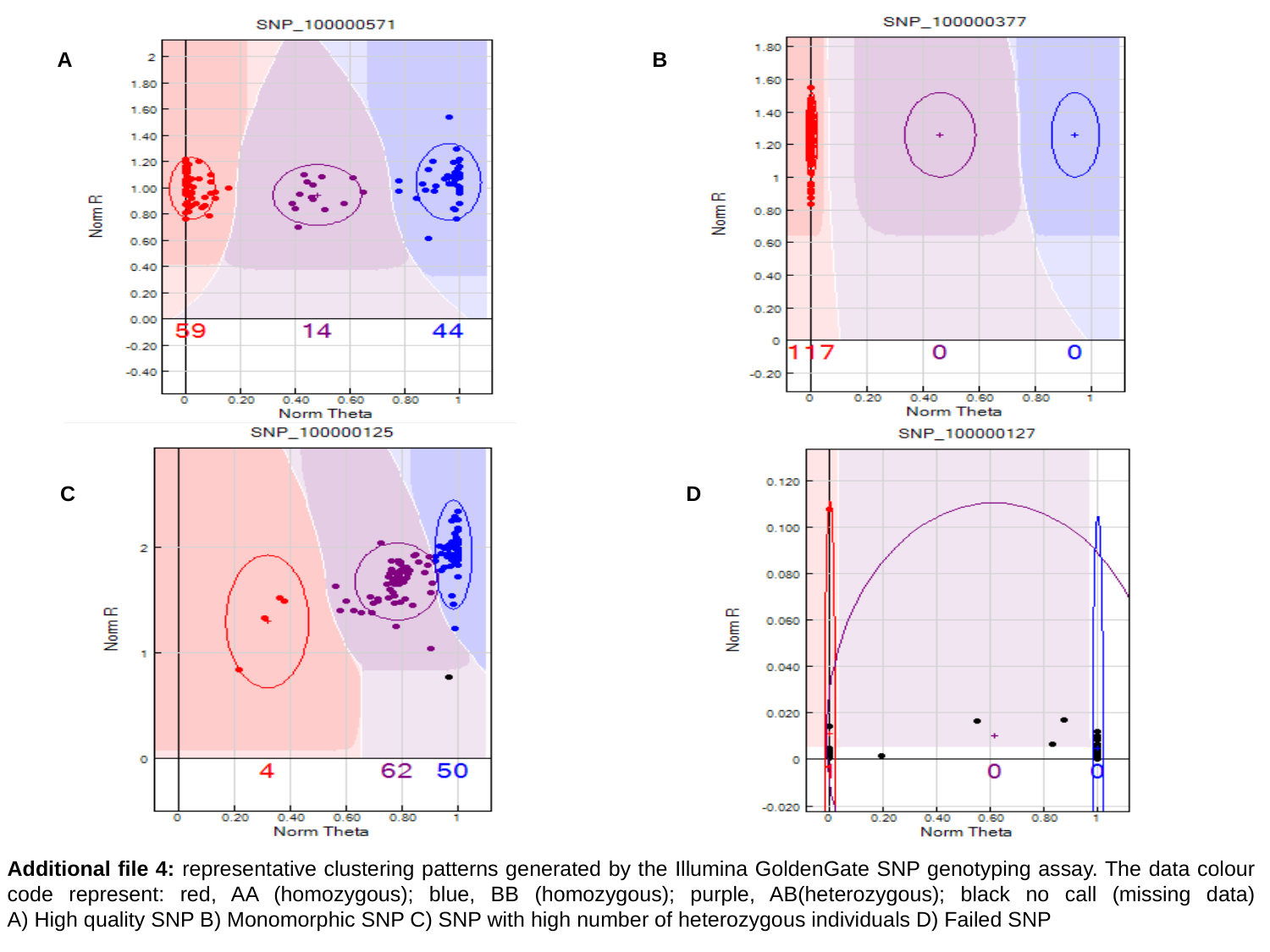

A
B
C
D
Additional file 4: representative clustering patterns generated by the Illumina GoldenGate SNP genotyping assay. The data colour code represent: red, AA (homozygous); blue, BB (homozygous); purple, AB(heterozygous); black no call (missing data)
A) High quality SNP B) Monomorphic SNP C) SNP with high number of heterozygous individuals D) Failed SNP
