# Supplementary material for: SNP marker discovery, linkage map construction and identification of QTLs for enhanced salinity tolerance in field pea (Pisum sativum L.)
Source: BMC Plant Biol. 2013 Oct 17;13:161. doi: 10.1186/1471-2229-13-161 (PMC4015884; doi:10.1186/1471-2229-13-161)
Supplement: Additional file 7 — Frequency distribution histogram. Frequency distribution for salinity index value and qualitative rating (T (tolerant), MT-T (moderately tolerant to tolerant), MS-S (Moderately sensitive to sensitive), S (sensitive), HS (high sensitivity) for Kaspa x Parafield RIL progeny following salinity treatment of 18 dS m-1. [file 1471-2229-13-161-S7.pptx]

## Slide 1
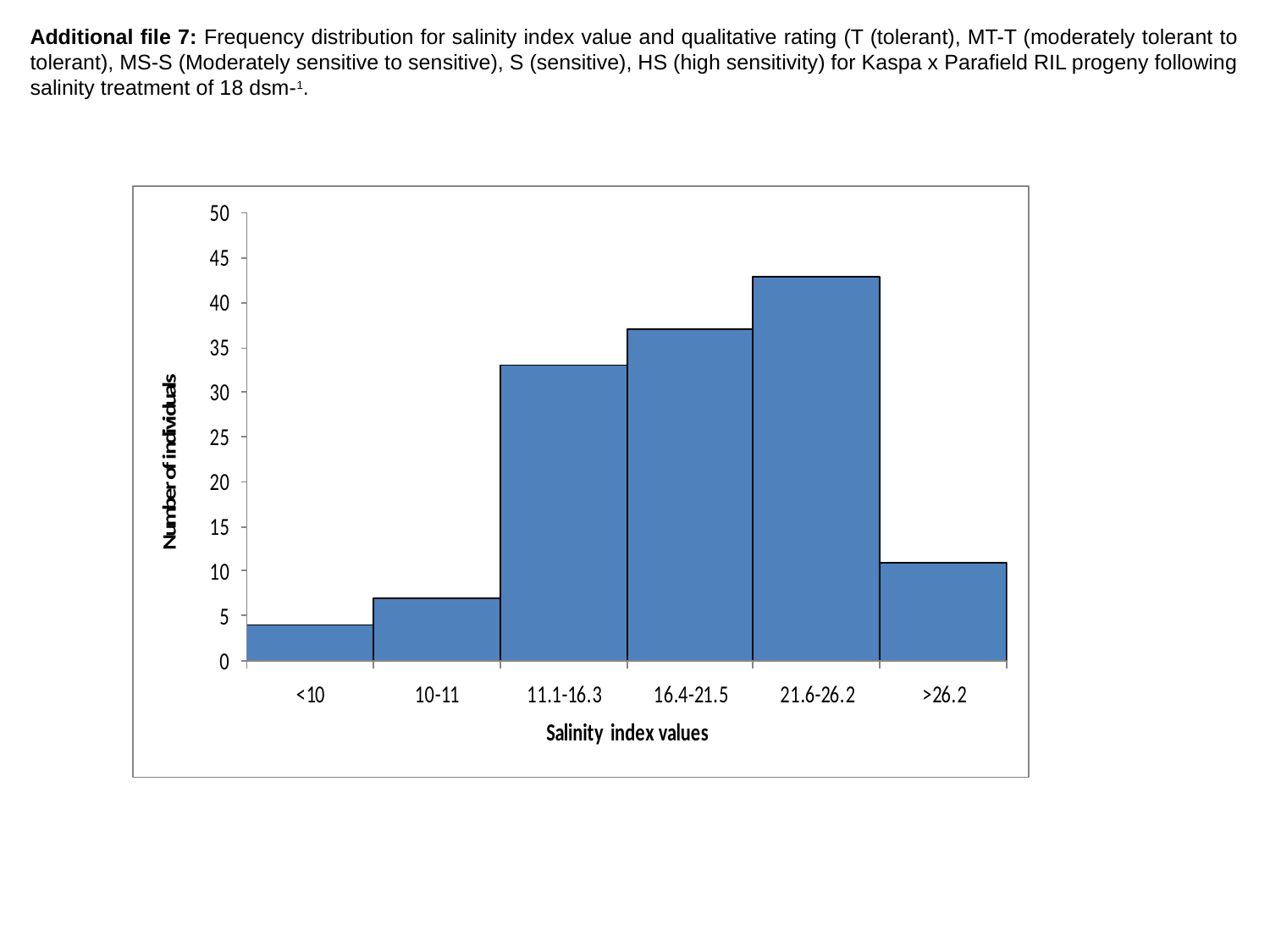

Additional file 7: Frequency distribution for salinity index value and qualitative rating (T (tolerant), MT-T (moderately tolerant to tolerant), MS-S (Moderately sensitive to sensitive), S (sensitive), HS (high sensitivity) for Kaspa x Parafield RIL progeny following salinity treatment of 18 dsm-1.
