# Supplementary material for: SNP marker discovery, linkage map construction and identification of QTLs for enhanced salinity tolerance in field pea (Pisum sativum L.)
Source: BMC Plant Biol. 2013 Oct 17;13:161. doi: 10.1186/1471-2229-13-161 (PMC4015884; doi:10.1186/1471-2229-13-161)
Supplement: Additional file 8 — Frequency distribution histogram. Frequency distribution for symptom score of Kaspa x Parafield RIL progeny at 7, 14, 21, 35, 42, 49 and 56 days post application of NaCl in watering solution at 18 dS m-1. [file 1471-2229-13-161-S8.pptx]

## Slide 1
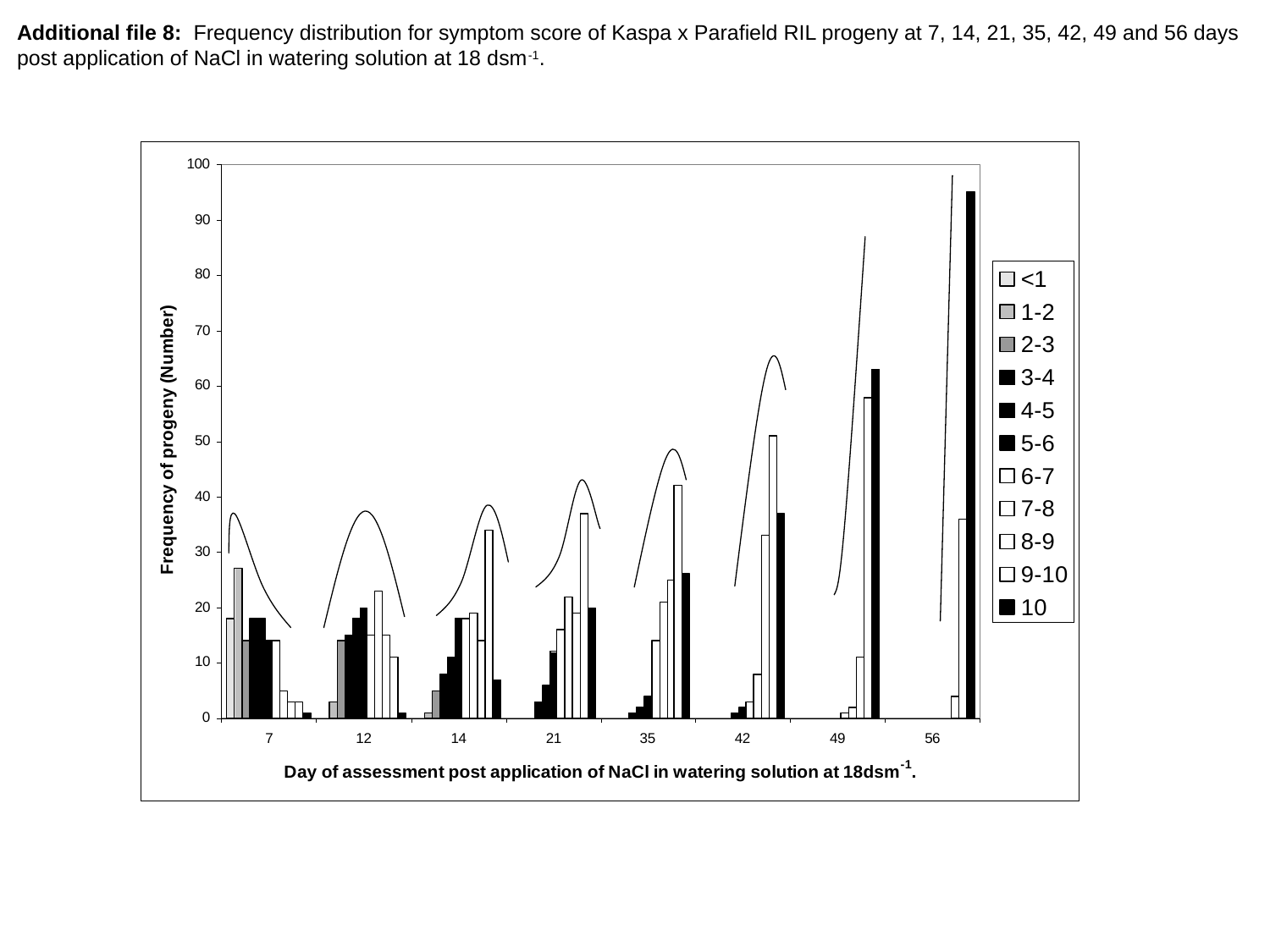

Additional file 8: Frequency distribution for symptom score of Kaspa x Parafield RIL progeny at 7, 14, 21, 35, 42, 49 and 56 days post application of NaCl in watering solution at 18 dsm-1.
